# Roboethics of tourism and hospitality industry: A systematic review

**DOI:** 10.1371/journal.pone.0287439

**Published:** 2023-06-30

**Authors:** Jinsheng Jason Zhu, Zhiyong Liu, Tairan Huang, Xue Shirley Guo

**Affiliations:** 1 Belt and Road International School, Guilin Tourism University, Guilin, Guangxi, China; 2 International Hospitality Management, Taylor’s University, Subang Jaya, Malaysia; 3 College of Business and Economics, The Australian National University, Canberra, Australia; 4 School of Hospitality Management, Guilin Tourism University, Guilin, Guangxi, China; 5 School of Hospitality, Tourism and Events, Taylor’s University, Subang Jaya, Malaysia; University of Donja Gorica, MONTENEGRO

## Abstract

This study aims to give a comprehensive analysis of customers’ acceptance and use of AI gadgets and its relevant ethical issues in the tourism and hospitality business in the era of the Internet of Things. Adopting a PRISMA methodology for Systematic Reviews and Meta-Analyses, the present research reviews how tourism and hospitality scholars have conducted research on AI technology in the field of tourism and the hospitality industry. Most of the journal articles related to AI issues published in Web of Science, ScienceDirect.com and the journal websites were considered in this review. The results of this research offer a better understanding of AI implementation with roboethics to investigate AI-related issues in the tourism and hospitality industry. In addition, it provides decision-makers in the hotel industry with practical references on service innovation, participation in the design of AI devices and AI device applications, meeting customer needs, and optimising customer experience. The theoretical implications and practical interpretations are further identified.

## Introduction

Many individuals believe that Industry 4.0 might be characterized by the increased adoption of networking technologies and intelligent automation in current organizations. An innovation paradigm for the advancement of technology seems to be developing [1, 2], one that incorporates automated machine learning [3, 4], deep learning [5, 6], nanodevices [7, 8], quantum physics [9–11], and self-driving automobiles [12]. As a result of developments such as technology and time/space compression [13, 14], how we communicate and cooperate in the future will be different. It is projected that both the capabilities and performance of artificial intelligence (AI) will continue to develop in the coming years, making it one of the technologies that are considered cutting-edge [15, 16].

AI was predicted by practitioners in the hotel business professionals that its application can enhance both the quality of services provided and the experiences provided to customers. They had high hopes that the AI they had implemented would be beneficial to their management and operations. Despite the fact that a growing number of hospitality organisations have adopted AI devices [17, 18], customers’ interest in and use of AI gadgets is lower than anticipated [19, 20]. Acceptance by users is the determining factor in the successful adoption of any brand-new technology [21]. To avoid losing money on AI investments and make the most of the opportunities presented by its use, experts in the hospitality industry need to investigate the factors that influence the acceptance and use of AI devices by customers [22].

To minimise unnecessary AI investments and maximise the potential benefits of AI incorporation, hospitality professionals should investigate factors that influence the acceptance and use of AI devices by customers. As more and more applications are found for artificial intelligence, researchers have begun paying a lot more attention to AI’s underlying difficulties. Initially, AI research was carried out by engineers, who mostly concentrated their efforts on AI design challenges [23]. These concerns included AI appearance, mapping, picture recognition, and other similar topics. A social science flavour has only recently been added to artificial intelligence research, which focuses on human-AI interaction, user perceptions, and acceptance of AI devices as service providers. This is a relatively new development, as social science researchers have only recently begun entering the field [24–29]. Research into artificial intelligence is still in its immaturity as a direct result of the comparatively short history of AI deployment. In artificial intelligence studies with a social science perspective, a significant amount of emphasis is focused on doing conceptual and descriptive research [27]. The development of a theoretical framework for the use of AI devices and the decision-making process was the primary emphasis of this research [30, 31]. These studies analysed previous work done in the field of artificial intelligence device implementation literature (such as research on service robots), with the objectives of providing an explanation for the phenomenon of AI adoption and identifying suitable future study fields [32]. The preponderance of research conducted to understand the phenomena of AI adoption has been conducted from the service provider’s perspective. These studies have mostly examined the usage and effects of AI devices on the operation and administration of service providers, including cost reduction [33], investments [34], workforce management [35, 36], as well as work environments [37, 38]. These results should be taken with an amount of caution since there hasn’t been a great deal of study on how people react to and employ AI devices.

### Research methodology adopted

**Fig 1:**
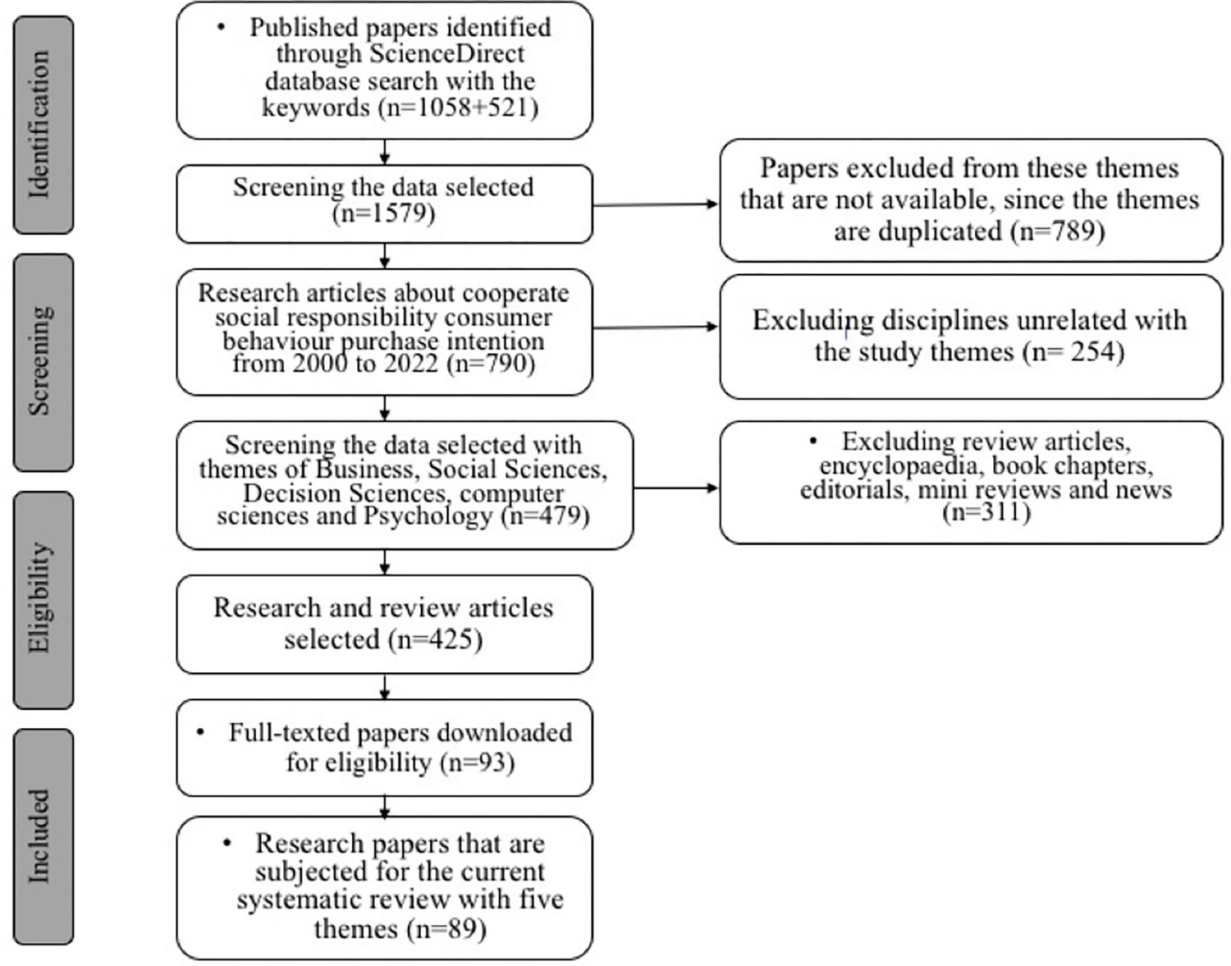
PRISMA research process. (Source: the authors’ own elaboration) (Keywords: human / computer / interaction / hospitality / tourism / ethics, each of the words shown in different research endeavour).

By focussing on a larger number of tourist and hospitality journals, the purpose of this investigation was to find a way around the constraint previously mentioned. Preferred Reporting Items for Systematic Reviews and Meta-Analyses, or PRISMA for short, is a further addition to this research endeavour [39]. In the field of medicine, this specific approach to systematic practice is extensively well-known and respected [40]. However, scholars and academics in the tourism industry rarely use it, except for some outstanding literature [41, 42]. In particular, this study investigates whether or not the PRISMA checklist items have been used in any systematic reviews that have been carried out in the field of tourism and hospitality by assessing the items on the checklist. Because of this, the author’s awareness of the methods that tourism academics use when doing systematic reviews has been one of the major contributions as a result of this study. This study will provide researchers with criteria for performing a suitable-practice systematic review and pave the route for the authors to use PRISMA in the current study. Importantly, this paper provides a complete analysis of the systematic evaluations that have been published in journals dealing with hospitality and tourism themes (see Fig 1 below). This underrepresentation of the tourism and hospitality themes was the primary impetus for the decision of delving into a such research topic. This current comprehensive analysis of review papers in this paper provides more illumination on a variety of domains of roboethics knowledge as an outcome of the research endeavour.

**Fig 2:**
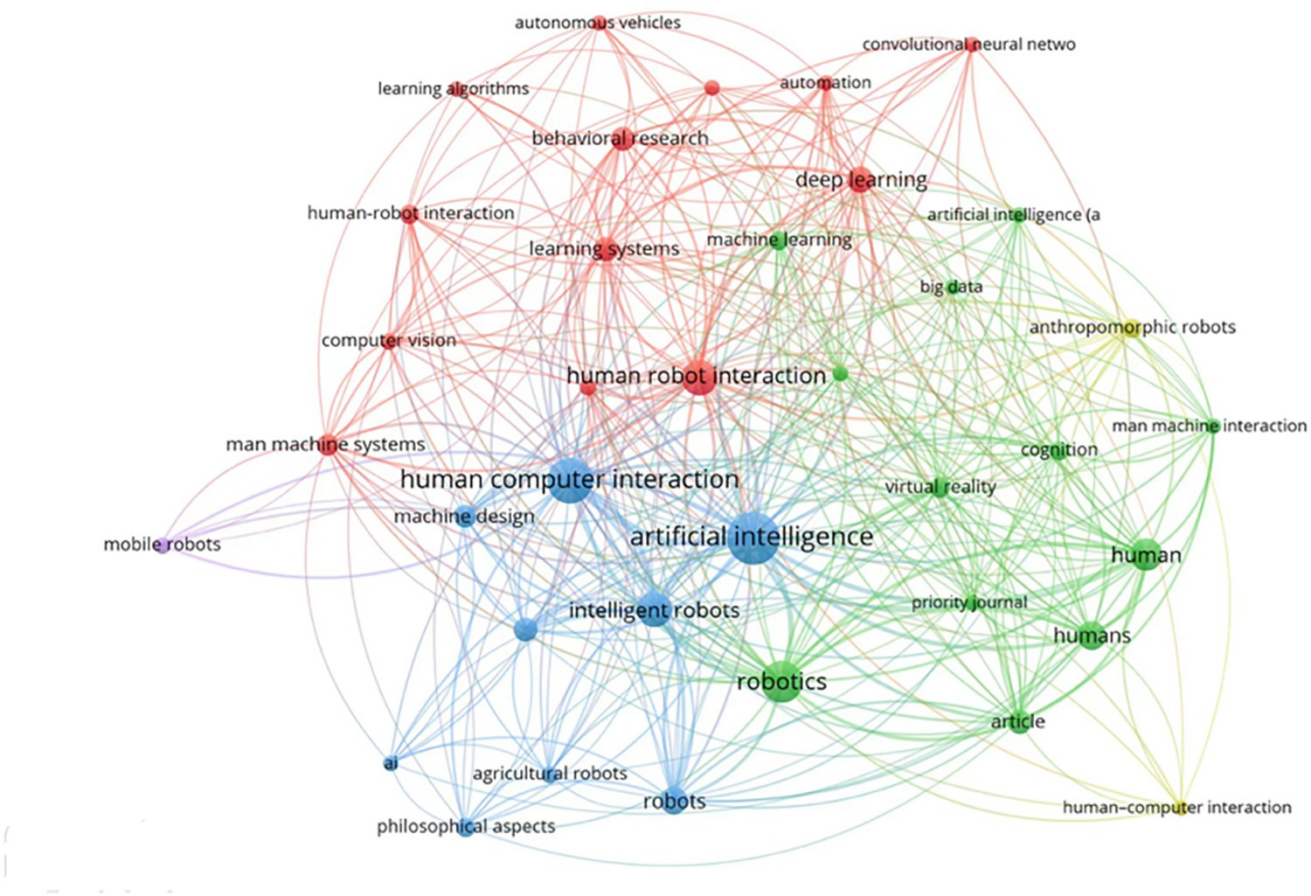
Keyword co-occurrence map. (Source: our elaboration from the VOSviewer software).

This systematic review followed the Preferred Reporting Items for Systematic Reviews and Meta-Analyses guidelines [43, 44]. The author conducted a detailed search for previously published systematic reviews that were included in hospitality and tourism journals. Using a dataset produced from this summary, this study was analysed to determine the level of quality of systematic reviews conducted in the hospitality and tourist industry. In the current study, the PRISMA reporting checklist parts that are utilized to carry out systematic reviews in the social sciences are broken down, and more explanation is provided [39, 45, 46]. A protocol was prepared in advance to record the analytic process and inclusion criteria for the primary dataset [47–49]. Web of Science, ScienceDirect.com and the journal websites were used to search for articles published in tourism and hospitality journals of high quality that had reviews in their titles, abstracts, and/or keywords relating to a systematic review, tourism, hospitality, AI, robot, ethic(s) and human-computer interaction (see Fig 2 below for its keyword co-occurrence frequency outcome). The paper selection criteria are listed as follows.

1. Selected data must be related to robot ethics-themed articles and reviews on the tourism and hospitality industry.
2. Sources other than the English language have been excluded in the systematic review of the current paper.
3. Papers not related to human-robot interactions are excluded.
4. There is no time limit for the selected papers in the current study.

For instance, since August 2022, the investigation has been continuing until the formal draught of the manuscript has been produced. The information that was found in the identified entries’ previously released articles was transferred to a database created in Microsoft Excel. The evaluation of the title and abstract was completed independently by the first author and the corresponding author of the current paper. Subsequently, the whole content of the selected papers was scrutinised in light of the qualification requirements. In particular, the author looked at studies that found relevant material by exploring several sources using a set of phrases that had already been determined.

**Fig 3:**
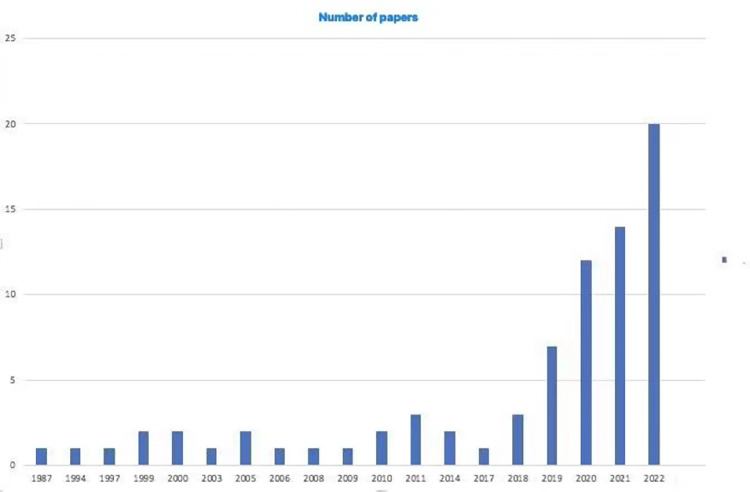
The number of published papers as per the publishing years. (Source: Authors’ elaboration).

Accordingly, the allocation of scientific publications per publishing year was subsequently constructed. Fig 3 depicts the progression of academic output on the specified subject across the whole period of time between 1987–2022. In the early phases of implementation, there were very few publications. From 1987 through 2017, a small number of papers were recorded annually, except for the year 2011, when three pieces were published. In the years between 2018 and 2022, the quantity of chosen topics grew rapidly and substantially. Regarding 2022, it needs to be understood that it is continuing, therefore, the 20-article data shown is not conclusive. Therefore, the increasing tendency of previous years and the substantial number of papers will continue to exhibit a culminating tendency of expansion. Human-computer interaction and robotethics concerns in the tourist and hospitality industry have attracted a growing amount of attention in recent years, as shown by a rising trend identifying the last few years as being increasingly more productive and diversified.

## Results

### Researchers’ attention to AI adoption

The term artificial intelligence (AI) is used to describe computer programmes that simulate human intelligence in judgment by combining complex software and hardware components with massive data [50]. Alternately, artificial intelligence (AI) can be understood as the capacity of a system to accurately interpret sensory stimuli, acquire new knowledge, and apply such knowledge learned to accomplish original objectives in an adaptive approach [51]. As more and more applications are found for artificial intelligence, researchers have begun paying a lot more attention to AI’s underlying difficulties [52–55]. Research on artificial intelligence (AI) was first carried out by engineers, who primarily focused their efforts on AI design challenges such as the appearance of AI, cartography, visual identification, and other similar topics [23, 55]. A sociology flavour has only recently been incorporated into computational intelligence, which concentrates on human and AI interplay, perceived utility, as well as the acquiescence of AI technologies as service suppliers. This is a relatively new development, as sociologists have only barely started entering the domain [20, 27, 28]. AI research is ongoing in its early stages as a result of the relatively shorter history of AI implementation in human history. The artificial intelligence research that took a social science perspective placed a strong emphasis on theoretical and implementation studies that are geared toward conceiving how AI devices are employed and how decisions are made [56, 57]. They described the phenomenon of AI interaction with AI-facilitated systems and proposed future research fields [58, 59]. These studies have focused primarily on the use of AI devices and the effects these devices have had on the management and operation of the service provider’s business, such as cost-benefit balancing [60], operational functions [61], job opportunities [62, 63], staff competencies enhancing [64], and facility designs [65–67], among others. The bulk of research conducted to understand the phenomena of AI adoption has been conducted from the service provider’s perspective, while these findings should be taken with a smidgen of scepticism since there has not been a great deal of study on how people react to and employ AI devices. There are three distinct ways that ethics might be interpreted in the robotics discipline [68, 69], which include the moral codes that are programmed in robotics, the ethics of individuals who build while using robots, and the morality of how people interact with automatons. The works of literature pertaining to robot ethics in service would be included in the systematic review in order to further delve into these ethical themes concerning the service business, particularly in the tourism and hospitality industry.

### AI applications in service areas

Despite the fact that conventional interactions between consumers and human employees continue to be the norm, artificial intelligence has gained prominence in recent years [70]. Artificial intelligence enables robotics to tackle issues in a manner equal to that of humans by incorporating human characteristics into complex algorithms. Consequently, using deep learning algorithms, artificial intelligence systems may be able to assist companies in creating customised offers based on prior client requests and preferences. AI is permeating several businesses, notably the service industries, because of its profit-enhancing potential and technical developments. As artificial intelligence technology has advanced and digital marketing has become more important, businesses in a wide variety of service industries, such as healthcare coverage, financial services, general merchandise, healthcare, youth development, commuting, tourist activities, and hotel management, have integrated AI devices into their service provision and operational processes [71, 72]. The devices with AI technologies can provide services in various ways. Opinion mining [73], which is propelled by AI technologies such as natural language processing, has the potential to discover and automatically assess the perspectives of travellers on the qualities of items sold in the tourism industry. Chatbots and messaging that are driven by AI are being used by firms that provide Smart Support to enhance both functional and technical support operations [74, 75]. Recommender systems that are powered by artificial intelligence are used to provide visitors with a list of activities that are tailored to their preferences and requirements [76]. Robots and service automation techniques such as delivery robots, chatbots, robot-concierges, and self-service kiosks have been utilized to enhance corporate performance and customer service in the hotel business [77, 78]. It has begun to get substantial attention as a result of the growth in the number of AI-powered solutions that are used in the service delivery process.

### Ethics issues of AI and roboethics

Lin et al. [79] outline the increasing presence of robotics in humanity, from safety to sexuality, and examine the myriad of ethical and societal difficulties. In the study, Lin classifies these issues into three key areas, such as security and faults, laws and ethics, and the interplay of social relationships. Malle [80] gives a framework that specifies what a normatively competent automaton would entail, which is commonly referred to as computer morality, in an attempt to more effectively merge the morals of robot operation in society. In the meantime, examine a variety of ethical concerns that reflect the deployment, utilization, and therapeutic interventions of such ethical androids in social structures. Such a topic is typically referred to as robot ethics. Meanwhile, Vanderelst and Winfield [81] present a strategy for programming robots with ethical conduct based on the simulation theory of cognition. As a philosophical study, it gives a theoretical perspective; nevertheless, no practical proposals are provided addressing which robots should bear responsibility in which situations or how rules for the responsible use of robots need to be created. The best approach to robot ethics, according to Asaro [69], is one that covers all of the ethical difficulties involved with robot ethics, and in order to do so, it must consider that robots are serving in a societal-technical system. Danaher [82] attempts to illustrate and expand the concept that robot processing is a guideline to the ethics of robotics by using prior work on robotic morality as a basis. Following these, Burton et al. [83] continue to give AI instructors and programmers case studies and linkages to service providers and service resources. While expressing and agreeing on ethical concepts for robots is essential, it is simply the beginning of comprehending the social structure of robotic ethics [84, 85]. Comparisons with the field of robot ethics shed light on some of the limitations of theories, including the fact that they are at times much too comprehensive and theoretical to interpret ethics in practice. In what is intended to be a quasi-examination, compare and contrast a wide range of ethical standards by highlighting both their similarities and their differences [86]. To get an understanding of the role that checklists play in AI ethics. It is important to concentrate on fairness while engaging in an iterative design process with a few AI practitioners. Siau and Wang [87] investigate the ethics of artificial intelligence by analysing AI ethics in modern times. Their principal innovation is a theoretical analysis of the field at a higher-level abstraction, in which they offer core concepts, outline methodology, and discuss critical concerns in AI ethics.

### Different views on AI adoption in the tourism and hospitality industry

As a result of the growing prevalence of artificial intelligence (AI) technologies and artificial intelligence gadgets within the hospitality sector, the customer and provider possess distinct points of view regarding the utilization of AI [75, 88–90]. From the point of view of service suppliers, artificial intelligence gadgets have the potential to enhance businesses’ productivity, effectiveness, and security reduced expenses, improve quality service, facilitate a sustainable workforce with synergic collaboration between human staff and AI-given service, and enhance employee satisfaction, thereby improving employees’ overarching well-being of all stakeholders in the tourism and hospitality businesses [91, 92]. Although some enterprises are aware that the initial investment necessary for AI technologies may be rather significant, they are nonetheless excited to incorporate these technologies within hospitality businesses [17, 93]. According to the uncanny valley theory [94, 95], which is a graphic depiction of the relationship between human people’s affinity and AI devices’ realism (i.e. similarity to humans), the devices’ realism increases as the degrees of human individuals’ affinity and acceptance for AI devices increase. In other words, the greater the capacity of people to accept and identify with AI gadgets, the more realistic they look.

Clients might well have conflicting opinions about the adoption and usage of AI gadgets in the hotel industry. On the one hand, some current existing researches suggest that AI gadgets may increase consumers’ perceptions of service excellence and reliability, hence increasing their acceptance of their usage in accommodation facilities [96, 97]. The usage of AI in the hospitality business may change the way that visitors evaluate and appreciate the services offered by hotels [23, 98]. It is indeed possible that AI devices will improve customers’ experiences. Customers are willing to accept and make use of AI devices in the hotel business [99]. On the other hand, however, the level of acceptance and use differs depending on the service that AI gadgets deliver. Not every customer is exhilarated by anticipating the bright future of AI devices. There is still a major crowd of customers, who continually show strong resistance to accepting and using AI devices in the hospitality industry. Customers who use AI gadgets express feeling isolated while using adopting computer-mediated communications as a consequence of the reduced opportunity for social connection, which ultimately results in their decision to forego the utilization of high-tech devices [100]. Customers perceive that the hospitality industry should place more of an emphasis on human value as opposed to robotic value [101, 102]. In particular, they believe that luxury hotels and hotels that do employ robotic services should place a greater emphasis on the experience guests have while staying at their properties.

Moreover, the perceived human-likeness perceived intelligence and perceived danger including privacy, safety, and security problems might influence consumers’ adoption and usage of AI gadgets in the hotel industry [103]. Some users believe that the humanlike characteristics of AI products might undermine their human identity [104]. When the level of realism of AI devices hits a certain limit, the affinity connecting humans and AI gadgets will decline substantially, resulting in unexpected feelings, or even perceptions of risks [105, 106]. Customers may be hesitant to adopt AI technology because of anxiety that humans may lose their individuality to humanoids, causing hotel technology resistance [107–109]. Many consumers who are unwilling to accept and employ AI technologies are also concerned about their privacy [110]. AI devices equipped with machine intelligence are regarded as an effective method for increasing the customer experience, since they utilize significant consumer data to anticipate their needs and tailor their services. Customers are reluctant to share or expose their personal information with hospitality service suppliers. When customers discover that service providers surreptitiously collect, store, and use their personal information, they may feel increasingly less secure. For instance, some customers may disapprove if a hotel monitors everything they do throughout their stay, including what customers watch on television and what people eat [111]. Individuals may feel worried about their privacy being violated, despite the fact that such information would be used to produce better-tailored customer service. Tourism and hospitality businesses and their clients will be affected by security breaches.

### Tensions in roboethics in the tourism and hospitality industry robot-adoptions

Customers’ perceptions of the adoption and utilization of AI gadgets vary from optimism over the enhancement of their experience to the anxiety of an automated society [15, 112]. In addition, existing technology acceptance theories have been used to investigate the acceptability of AI gadgets by consumers and their employment in service settings [113–115]. Because these theories were designed to explain the acceptance and usage of non-intelligent technology gadgets, several researchers suggested that they may not be suitable to investigate consumers’ acceptance and use behaviour in the setting of AI devices [116, 117]. Since AI devices have unique intelligent natures that are significantly different from non-intelligent technologies devices such as humanlike mindsets that require fewer customers’ learning to operate the devices, which makes the ease of use as core constructs in traditional technology acceptance models irrelevant to explain the drivers of customers’ acceptance and use of AI devices [118].

In addition, the previous study has investigated the acceptance and use of AI devices by customers in a variety of service settings [119, 120]. According to the findings of previous studies, the varying degrees of services and the nature of utilitarianism and hedonism may vary from one service to another [121, 122]. This difference has the potential to have a significant impact on the expectations of consumers and the hotel products the consumers intend to purchase. Customers in the hospitality business seem to have higher expectations of hedonic value (that is, enjoyable and unique experiences) than those in other service industries. Additionally, in comparison to other types of organizations, the hospitality sector has far more frequent interactions between customers and employees [123].

### Roboethics in tourism and hospitality amid the COVID-19

**Fig 4:**
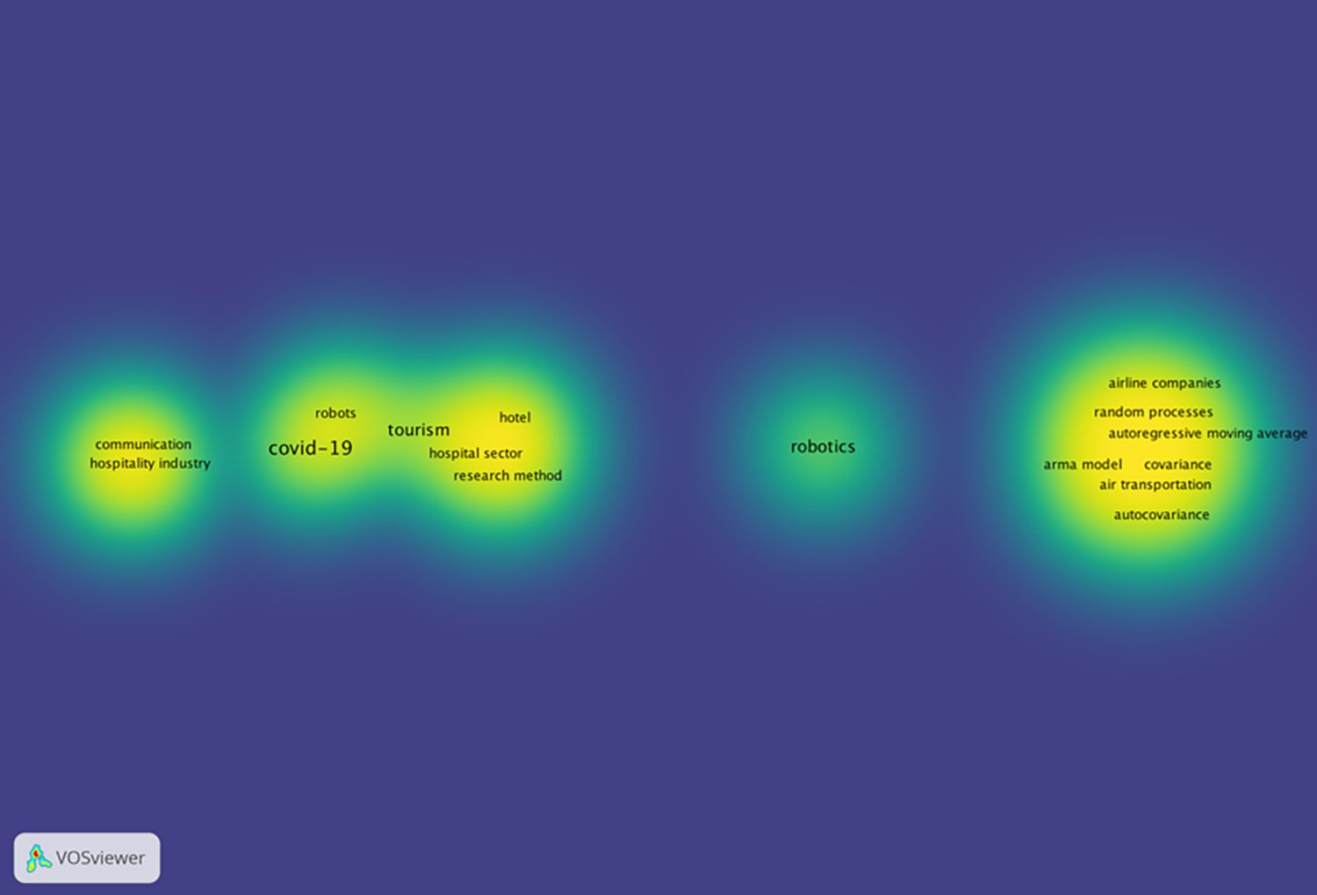
Keywords (robot AND tourism OR hospitality AND COVID) density map. (Source: our elaboration).

Coronavirus disease 2019 (abbreviated as COVID-19) is swiftly disseminated over the globe through human pathogens [124–126]. The pandemic has prompted a massive worldwide public health push to reduce social interactions and increase clear distance. Numerous ideas demonstrate that unpredictability and poor consistency not only endanger people’s choices of physical health, but also their mental health, particularly in the psychological and cognitive domains (see Fig 4 for the keyword co-occurrence map). After a crisis, customers’ conduct will alter [127–129]. Previously, researchers have observed that customers’ acceptance and use of AI devices in the hospitality business are not promising due to the customers’ desire for personalized amenities with actual staff members [130–132]. History demonstrates that technological innovation and advancement may aid in disaster or crisis management [133]. For instance, robots were used in the 2011 Fukushima nuclear plant disaster [134]. In three major areas, including health treatment, logistics, and reconnaissance, robotics may aid the pandemic [135]. In hospitals, airports, transportation systems, recreation and scenic areas, hotels, restaurants, and communities in general, AI devices such as robots, autonomous vehicles, and drones have played a significant role in managing the potential spread of COVID-19 [136, 137]. They are responsible for delivering items, disinfecting and sterilizing public spaces, detecting or measuring body temperature, providing safety or security, and comforting and entertaining patients and customers [133]. The quick growth of robots, automation, and artificial intelligence (AI) is anticipated to impact and revolutionize many facets of the hotel and service sectors, particularly after COVID-19.

When the COVID-19 pandemic is still underway, the public has also been made aware of the advantages that AI gadgets may provide in terms of facilitating the preservation of social distance and minimizing the danger of infection. During the COVID-19 public health disaster, it is probable that hotel consumers may be increasingly keen to utilize AI devices [138, 139]. Customers may utilize self-service technology to check in or check out, get service information through chatbots, enjoy hotel amenities offered by service robots, and modify the room’s amenities via smart systems. In light of the fact that the production of the vaccine has not yet reached full maturity, there is an urgent need to conduct an empirical study to determine how customers’ perceptions of the threat posed by COVID-19 affect their willingness to accept and utilize AI devices in the hospitality industry. Due to the extremely infectious nature of COVID-19, maintaining a safe social distance between individuals has proved to be an effective method of preventing viral infections [140, 141]. The pandemic has prompted a massive worldwide public health push to preserve social distance by reducing direct human-to-human contact and high physical distances. These practices of social distance have had a significant influence on businesses that depend heavily on human connection, such as hospitality and tourism, which are suffering severely during this era [142, 143].

## Discussion and conclusion

This study employed a systematic review following the PRISMA guidelines to provide a comprehensive knowledge of AI adoption in the hospitality industry and its relevant ethical issues. According to the PRISMA guidelines, the inclusion criteria and data collection process are explained. The present research examined 89 relevant research articles from prestigious databases such as Web of Science and ScienceDirect.com, as well as journal websites. The paper presents a keyword co-occurrence map and the number of published papers per publishing year to provide an overview of the AI research papers’ landscape in hospitality. The study identified six research domains related to the publication themes, highlighting the advantages and complexities of AI technology in the hospitality industry. It summarizes the applications of AI in service areas and discusses different views on AI adoption from the perspectives of service providers and customers in the tourism and hospitality industry. Furthermore, it also references various studies that have explored the ethical implications of AI in the hospitality industry. The ethical issues related to AI adoption such as resistance by employees, competition with rivals, and legal issues are identified. which are essential and not frequently raised in publications. The paper contributes to the existing literature by providing a comprehensive analysis of AI adoption in the hospitality industry and emphasizing the need for further research in understanding the roboethics issues for AI adoption. The insights gained from this study can help hospitality professionals make informed decisions regarding AI investments and ensure the optimal utilization of AI technologies in their operations.

### Theoretical implications

The literature review included in this study suggests that the tourism and hospitality-related publications have developed in terms of not only a rise in volume but also a growing diversity of topics. However, significant research gaps and under-researched areas in the tourism industry were also revealed. Future studies should investigate more complicated smart environments in which robots interact simultaneously with other robots and people, as they become more autonomous and interconnected with the Internet of Things (IoT). In addition, interdisciplinary research collaborations are required to provide more robust and widespread research on AI technology. Future studies on human concerns should include replication studies to examine the effects of robots on the tourism and hospitality experience and the attitudes, requirements, and hopes/fears of staff. The integration of robots into the behaviours of customers and service staff in the tourism and hospitality industry should be examined concerning morality and ethics.

### Practical implications

This research provides managers and marketers in the hotel industry with essential information to establish appropriate AI device investment and adoption strategies. It aids in increasing their understanding of consumers’ motivations for utilizing AI devices, proposing business strategies for planning, operating, and marketing their businesses, and enhancing customer experience using AI devices. It also enables hotel managers to strike a balance between the increased value-added requests of consumers, the technological advancement of the business, and the high danger of disease transmission.

### Limitations

The present research has two limitations. First, the theoretical framework and research findings used in this study are restricted to the present era. Second, the data collection approach will consist of conducting a systematic review of a larger base of hotel research to determine their acceptance and usage of AI devices based on their views. Thus, the findings may vary significantly if the sample consists of actual hotel guests who have stayed in specific hotels that offer service through AI gadgets.

### Future research implications

As AI technology rapidly advances, customers’ adoption and usage of AI products may alter drastically in the near future. Therefore, it will be important to develop a theoretical framework that encompasses the nature of AI variables in the future to predict the factors that impact consumer acceptance and use of AI devices and the relevant ethical issues that AI created should be laid stress by future research.
